# Comparing Methods to Impute Missing Daily Ground-Level PM_10_ Concentrations between 2010–2017 in South Africa

**DOI:** 10.3390/ijerph18073374

**Published:** 2021-03-24

**Authors:** Oluwaseyi Olalekan Arowosegbe, Martin Röösli, Nino Künzli, Apolline Saucy, Temitope Christina Adebayo-Ojo, Mohamed F. Jeebhay, Mohammed Aqiel Dalvie, Kees de Hoogh

**Affiliations:** 1 Department of Epidemiology and Public Health, Swiss Tropical and Public Health Institute, Socinstrasse 57, CH-4002 Basel, Switzerland; oluwaseyiolalekan.arowosegbe@swisstph.ch (O.O.A.); martin.roosli@swisstph.ch (M.R.); nino.kuenzli@swisstph.ch (N.K.); apolline.saucy@swisstph.ch (A.S.); temitope.adebayo@swisstph.ch (T.C.A.-O.); 2 Faculty of Science, University of Basel, CH-4003 Basel, Switzerland; 3 Centre for Environmental and Occupational Health Research, School of Public Health and Family Medicine, University of Cape Town, Rondebosch, 7700 Cape Town, South Africa; mohamed.jeebhay@uct.ac.za (M.F.J.); aqiel.dalvie@uct.ac.za (M.A.D.)

**Keywords:** air pollution, Random Forest, imputation, particulate matter, environmental exposure, South Africa

## Abstract

Good quality and completeness of ambient air quality monitoring data is central in supporting actions towards mitigating the impact of ambient air pollution. In South Africa, however, availability of continuous ground-level air pollution monitoring data is scarce and incomplete. To address this issue, we developed and compared different modeling approaches to impute missing daily average particulate matter (PM_10_) data between 2010 and 2017 using spatiotemporal predictor variables. The random forest (RF) machine learning method was used to explore the relationship between average daily PM_10_ concentrations and spatiotemporal predictors like meteorological, land use and source-related variables. National (8 models), provincial (32) and site-specific (44) RF models were developed to impute missing daily PM_10_ data. The annual national, provincial and site-specific RF cross-validation (CV) models explained on average 78%, 70% and 55% of ground-level PM_10_ concentrations, respectively. The spatial components of the national and provincial CV RF models explained on average 22% and 48%, while the temporal components of the national, provincial and site-specific CV RF models explained on average 78%, 68% and 57% of ground-level PM_10_ concentrations, respectively. This study demonstrates a feasible approach based on RF to impute missing measurement data in areas where data collection is sparse and incomplete.

## 1. Introduction

Ambient particulate air pollution is a major environmental risk to health. An estimated 4.14 million mortality in 2019 was associated with exposure to ambient air pollution [1]. Routine ambient air quality measurements at a sufficient spatial and temporal scale are essential for the management and evaluation of ambient air pollution regulations, policies and mitigation measures. They are also crucial for calibrating air pollution statistical models for accurate exposure assessment in epidemiological studies investigating the link between air pollution and health. However, in low- and middle-income countries (LMIC), routine air pollution monitoring stations are sparse due to the limited financial, human and technical capacities to manage these monitoring networks [2,3]. The lack of air pollution measurements in LMIC obstructs the development of aforementioned air pollution models for estimating ambient air pollution exposures and thus informing population health studies. 

Particulate matter less than or equal to 10 micrometers in aerodynamic diameter (PM_10_ µg/m^3^) is associated with acute and chronic adverse health outcomes and it is of high public health significance globally [1,4,5]. PM_10_ is one of the criteria air pollutants in most countries including South Africa, it is measured in South Africa by an air quality monitoring network managed by three levels of government (National, Provincial and Metropolitan/Local) and privately managed air quality monitoring stations [6]. However, due to limited air quality management capacities, these monitoring stations are concentrated around the designated air pollution priority areas. To date, four areas (located in four of the nine provinces) of South Africa have been designated an air pollution priority area; Vaal triangle, Highveld, South Durban Basin and Waterberg based on historical evidence of poor ambient air quality due to the presence of possible source of air pollution [7]. The quality of available data is a major concern with only a small number of South Africa’s ambient air pollution monitoring stations accredited by South African National Accreditation System [8]. 

In South Africa, air quality measurements are often missing due to various reasons such as vandalization of monitoring facilities, and periodic interruption of measurements due to electrical shut down or breakdown of monitoring equipment. This has led to a significant number of monitoring stations being out of operation for months or years resulting in long time-series of PM_10_ measurements missing [9]. Inconsistent air quality data hampers epidemiological studies in South Africa from investigating the association between air pollution and health. Previous studies in South Africa have documented the trends in air pollutants for raising public health awareness about the need for air pollution control [10,11,12,13]. 

Univariable methods of unconditional mean or median, nearest neighbour have been compared with multivariable methods from regression models using other environmental predictors’ for imputing daily PM_10_ measurements [14,15]. Multivariable methods were reported to be more robust in performance when the proportion of missing data are higher than 10% [14]. The relationship between PM_2.5_ and PM_10_ at co-located monitoring sites was explored using multivariable methods with the aim to predict PM_2.5_ at sites with PM_10_ data only in Switzerland and India [16,17]. However, this approach is not feasible in South Africa due to the paucity of PM_2.5_ data as it was only designated a criteria air pollutant in 2012 [18]. 

Random forest (RF)—a machine learning method can be classified as a multivariable method that aggregates the predictions of several regression trees to improve the performance of single regression models. Several studies have been published using RF and other multivariable models to predict missing air pollutants in areas with no or sparse monitoring networks [16,17,19,20,21]. However, this study aims to leverage on the spatial and temporal dependence characteristics of air pollutants [22,23], by combining observed PM_10_ data with spatial and temporal predictors as well as chemical transport estimates of PM_10_, ozone (O_3_) and nitrogen dioxide (NO_2_) in a RF model to predict missing daily PM_10_ observation in some monitoring stations across four provinces of South Africa for years 2010–2017. The result of this analysis will be subsequently used to construct models to predict PM_10_ in areas without monitoring sites. 

## 2. Materials and Methods

### 2.1. Methods

The RF machine learning method was employed to accommodate the non-linear relationship between PM_10_ measurements and covariates. For each year we constructed RF models at 3 geographical scales to predict missing daily PM_10_ data: (1) one national model, using all daily PM_10_ measurements from the four provinces combined; (2) four provincial models using daily PM_10_ monitoring measurements from sites within each province; and (3) site specific models exclusively using daily PM_10_ measurements from individual sites. 

### 2.2. Monitoring Sites

**Figure 1:**
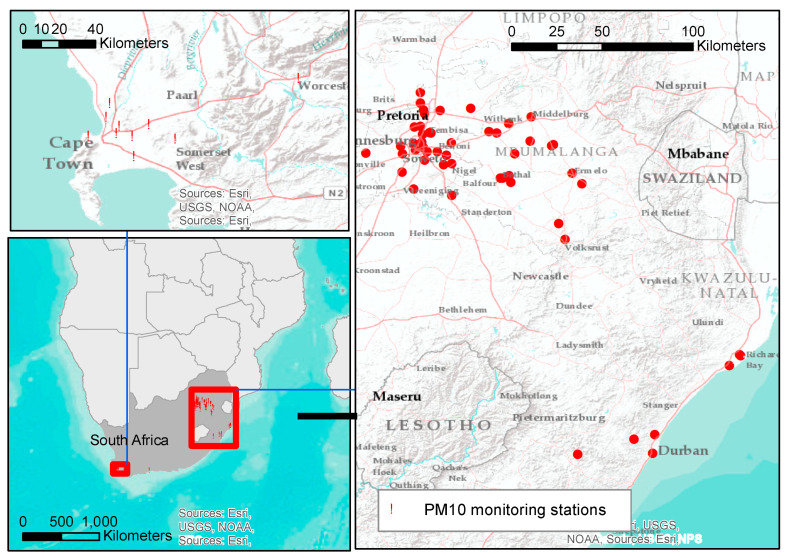
The spatial distribution of particulate matter (PM_10_) monitoring stations across the four provinces of South Africa operating at some point during 2010–2017.

**Figure 2:**
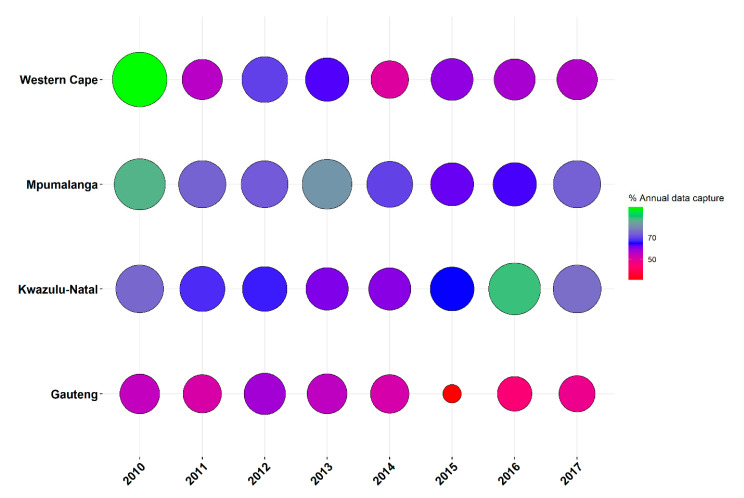
PM_10_ data availability by year and by province—the size and colour of the circles indicate percentage of data capture per year.

The focus of this investigation was on PM_10_ monitoring sites in South Africa, which are located in Mpumalanga, Gauteng, Western Cape and KwaZulu-Natal (Figure 1). These stations are managed by the Department of Environmental Affairs, South Weather Services, provincial, local governments and private industries. Hourly PM_10_ data from the four provinces were obtained from the South African Air Quality Information System (SAAQIS) for 61 monitoring sites (27 in Gauteng, 17 in Mpumalanga, 10 in Western Cape, 7 in Kwazulu-Natal) for the study period 1 January 2010–30 December 2017. Air quality monitoring stations instruments were serviced and calibrated bi-weekly, undergoing a full calibration annually, using National Metrology Institute of South Africa certified gases. The number of sites per year varies across the study period. Figure 2 shows the data completeness of the PM_10_ observations obtained from the SAAQIS by province between 2010 and 2017. SAAQIS provides PM_10_ data for research purposes in South Africa upon completion of the required data disclosure forms. SAAQIS can be reached via their website (https://saaqis.environment.gov.za/. Accessed on 22 October 2018).

### 2.3. Quality Check and Data Management

To ensure quality of the PM_10_ data, the following quality check filters were applied. All negative values or observations greater or less than four times the interquartile range of each monitoring stations were considered outliers and were subsequently removed. A threshold of 75% hourly data per day was used to aggregate hourly data to a daily mean concentration.

### 2.4. Temporal Parameters

Daily meteorological parameters of total precipitation, boundary layer height, temperature, the component of the horizontal wind towards east (U wind component) and the component of the horizontal wind towards north (V wind component) at a spatial resolution of 0.125 × 0.125° (approximately 10 × 10 km^2^) for the hour 12:00:00 were downloaded from the European Centre for Medium-Range Weather Forecasts Reanalysis 5th Generation (ERA5) global climate reanalysis dataset for the year 2010–2017 for South Africa. The U and V wind components were subsequently used to calculate wind speed (ws) and wind direction (wd) respectively using the formulas below:

(1) $$\mathrm{wd}=\;a\;tan\;2\left(-u_{10},\;-v_{10}\right)\times \;\frac{\pi }{180}$$

(2) $$\mathrm{ws}=\;\sqrt{u_{10}{}^{2}+v_{10}{}^{2}}$$

In addition to Copernicus Atmosphere Monitoring Service (CAMS) Reanalysis PM_10_ estimates, columnar daily ensemble estimates of pollutant gases of nitrogen dioxide, ozone were also downloaded from the CAMS data store at 0.125 × 0.125° (approximately 10 × 10 km^2^). All temporal predictors were resampled at a 1 × 1 km^2^ resolution, matching the 1 × 1 km^2^ resolution of the raster specifically constructed for this study. The monitoring stations locations were subsequently linked to this raster to extract the temporal predictors. 

### 2.5. Spatial Parameters 

A number of spatial geographic information system (GIS) predictor variables were calculated for this study at the aforementioned 1 × 1 km^2^ grid (see Table 1). South Africa’s road network was obtained from OpenStreetMap. For each 1 × 1 km^2^ grid cell, we calculated the sum of road length for two categories: major roads and other roads. Land cover data were extracted from the 2018 South Africa National Land cover dataset. The initial 72 land use classes were re-categorized into five major categories: residential; industrial; built-up; agriculture; and water bodies. South Africa’s climatic zones were extracted based on the South Africa Bureau of Standards 2005 classification. Population density was obtained from the Socioeconomic data and Application Center (SEDAC) dataset. For the light at night, data extracted from the Visible Infrared Imaging Radiometer Suite-Day/Night Band (VIIRS-DNB) was extracted and averaged at the 1 × 1 km^2^ resolution. Elevation and impervious surface were extracted from respectively the Shuttle Radar Topography Mission Digital Elevation Database version 4.1 and the National Oceanic and Atmospheric Administration database.

### 2.6. Random Forest Model

RF is a non-parametric machine learning algorithm and an ensemble method that can be used to perform regression for continuous outcome variable (e.g., PM_10_). Imputation of missing daily PM_10_ data for stations with at least 70% of annual PM_10_ was achieved by combining the measured PM_10_ and spatial and temporal predictor variables at three geographical scales; national, provincial and site specific.

To impute missing PM_10_, all possible monitoring stations with valid PM_10_ measurements were included in RF analysis. RF was used to estimate the PM_10_ concentration for the missing days by exploring the relationship between observed PM_10_ and spatial and temporal predictors. RF leverages on averaging several independent bootstrap ensemble trees to reduce the variance in the predicted PM_10_ by [24,25]:

1. Randomly resample the data with replacement to create training and validation sets of same sample size as the original dataset.
2. Repeatedly construct regression trees on the training sets and predict on the validation sets.
3. At each trees node, the best predictors from the random subsets of predictors were subsequently used to partition the nodes of respective trees.
4. The final estimate of PM_10_ is the average of individual trees of PM_10_ predictions in a process called bagging.

In this study, the RF parameters number of variables randomly sampled as candidates at each split (mtry) and number of trees to grow (ntree) and minimum number of observations in a terminal node (min.node.size) were selected based on the combinations that minimized out of bag prediction error in the one-third sample left out for validation. Throughout this study, 500 trees were considered. Generally, mtry was tuned at each terminal nodes with two and respective predictors to de-correlate the trees. RF models are less sensitive to parameter tuning for low dimensional data [26]. Similarly, using minimum number of predictors that substantially contribute to explaining the variance in PM_10_ could prevent overfitting the models as RF is prone to overfitting when spatial and temporal variables are included as predictors [27,28]. 

The feature importance of the models was ranked based on predictors that reduced prediction error when used as splits over the ensemble trees in the RF models. For all the RF models, the faster implementation of RF via the ranger packages was accessed from the caret package in R [29]. 

### 2.7. Model Validation

Spatial and temporal cross-validation was used to assess the daily PM_10_ models prediction errors in time and space. Spatial leave one location out cross-validation (LOLO CV) was used to evaluate the national and provincial models. The national model was split into four folds using the province as splitting criterion. Thus, a model was trained on data from all but one province (n − 1). The hold-out provinces sites were iteratively used to estimate the prediction errors of using these models to predict for sites not included in the training data. Sites were used as the splitting criterion for the different provincial models. To account for possible spatial autocorrelation in the models, a complete time-series of observations of a site was sequentially withheld (n − 1) for cross-validation. Spatial LOLO CV was not possible for the site-specific models. Temporal leave time out cross-validation (LTO CV) was used to assess the model’s performance in time. Day of the year was used to split the dataset 10 fold. All three models were sequentially trained on all but one held-out fold. All the models cross-validation were implemented using CAST (Caret Applications for Spatial-Temporal Models) package—a caret package wrapper for spatial and temporal cross-validation [28]. 

### 2.8. Error Metrics

Coefficient of determination (R^2^), the square of the correlation coefficient between the observed and predicted daily PM_10_ observation was used to evaluate the variance explained by the models. For all the models but sites models, we computed three R^2^ measures to assess the models performance. The model building R^2^ describing the overall models ability to explain the variance between observed and predicted daily PM_10_ observation. Spatial and temporal R^2^ to quantify the contribution of the spatial and temporal level to the total variance of daily PM_10_ model predictions on held-out stations and days. 

Root mean squared error (RMSE), the square root of the mean quadratic differences between observed and predicted daily PM_10_.

Mean absolute error (MAE), the average over the absolute differences between the observed daily PM_10_ and predicted daily PM_10_ were also calculated to provide summary estimates of the models prediction errors.

## 3. Results

### 3.1. National Model

**Figure 3:**
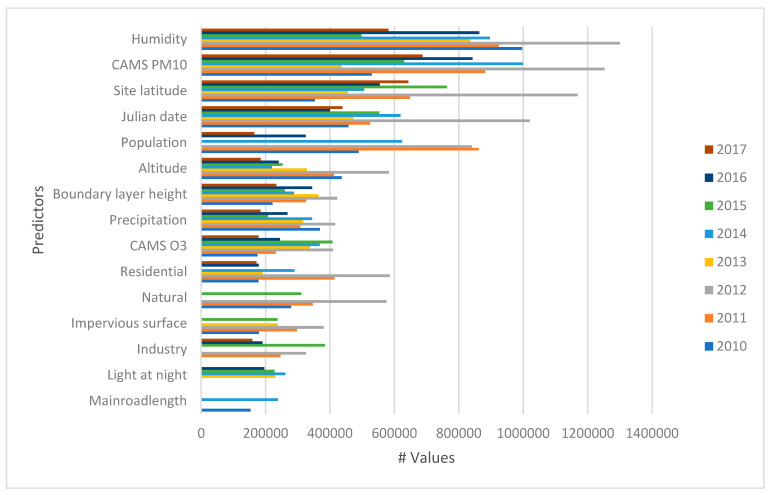
National model variable of importance.

The RF models combined spatial and temporally predictor variables with ground monitored PM_10_ from all the four provinces to construct national models for 2010–2017 (Table 2). Figure 3 shows the top 15 ranked variable of importance based on the predictors that reduced prediction error when used as splits over the ensemble trees in the RF models. Temporal predictors of chemical transport model-based estimates of PM_10_, humidity, Julian date and the spatial variable population emerged as influential variables across 2010–2017. The national RF models for 2010 to 2017 explained between 77% and 79% of the variation in daily PM_10_ concentrations. Spatial CV was used to assess the robustness of the models. The R^2^ of the spatial and temporal cross validation varies between 0.11 and 0.35 (RMSE: 17.72–29.47 µg/m^3^) and 0.77 and 0.79 (RMSE 12.31–16.43 µg/m^3^), respectively.

### 3.2. Provincial Model 

The provincial model explored the relationship between PM_10_ and predictor variables by each province across 2010–2017. Supplementary Material Figures S1–S4 highlight chemical transport model-based estimates of PM_10_, humidity, total precipitation, sites coordinates as variables of importance for explaining the intra-province PM_10_ variability. The contribution of these variables also varied across the study period and provinces—underlying the heterogeneity in the provincial characteristics of PM_10_ concentration. 

The performance of the provincial models while predicting PM_10_ for held-out sites varied across provinces and study period (Table 2). The CV results of the RF models for Gauteng, for example, explained between 26% and 52% of spatial variability and between 52% and 79% of temporal variability in measured PM_10_ concentrations. Mpumalanga RF models slightly improved on the Gauteng models with R^2^ ranges of 0.39–0.69 (spatial) and 0.73–0.78 (temporal). 

### 3.3. Site-Specific Models 

Site-specific or individual site models were used to assess the relationship between PM_10_ and temporal predictor variables if the site have at least 70% annual PM_10_ data. The site-specific models were explored independently from each other. The models for Witbank monitoring station performed best with explaining PM_10_ variability between 72% and 83% (Table 2). Leandra monitoring station performed worst with a range of explained PM_10_ variability between 29% and 36%. The temporal variables of chemical transport model-based estimates of PM_10_, humidity, Julian date, wind speed, temperature, total precipitation are important variables for explaining PM_10_ variability of the different sites (Supplementary Material Figure S5).

### 3.4. Models Prediction

Table 3 compares the distribution of observed PM_10_ values against the CV predicted PM_10_ for the three models (national, provincial and site-specific) for days with PM_10_ measurements. The site-specific models outperformed the national and provincial models in capturing the variability in PM_10_. The mean and the standard deviation of the predicted PM_10_ from the provincial and site-specific models are somewhat comparable to that of the observed PM_10_ concentrations. The range of the predicted mean PM_10_ concentrations from the national models differs substantially from the observed PM_10_ concentrations. 

## 4. Discussion 

This study explored methods for imputing missing daily PM_10_ measurements in South Africa, while considering the spatial distribution pattern of the sparsely PM_10_ monitoring stations across four provinces of South Africa. The RF models, representing three different geographical domains, exhibit markedly different predictive performances for predicting missing daily PM_10_ measurements across four provinces of South Africa.

The performance of the national models and provincial models decreased considerably when used to predict daily PM_10_ in the LOLO validation. Table 3 indicates that the provincial and site-specific models predicted PM_10_ concentrations do not differ substantially from the observed PM_10_ concentrations in terms of mean and standard deviation. In addition, we constructed a national model for the entire eight years (2010–2017) to compare the performance of this model to the yearly models (not presented in Table 2). The overall performance of the model R^2^ of 0.67 (RMSE, 17.70) suggest a reduced performance when compared to the range of the yearly models R^2^ of 0.77–0.79 (RMSE 2.10–16.76). The cross-validated spatial R^2^ of 0.24 (RMSE, 23.47) is within the range of yearly models R^2^ (0.11–0.35), RMSE (17.72–29.47). The better performance of the yearly models might be because most of the PM_10_ sites did not provide measurements consistently through the eight years. Also, the levels of PM_10_ between the years are different due to changing PM_10_ related emission variables. The national model, despite high overall R^2′^s (0.77–0.79), performed poorly in the LOLO CV (R^2^ 0.11–0.35). This was also reflected in the poor ability to predict the observed PM_10_ concentration (Table 3). This is perhaps not surprising given the large geographical domain of South Africa. The distances between the provinces are substantial (e.g., approximately 1000 km between Western Cape and the other three provinces) and, therefore, they exhibit different local emission characteristics driven by social and economic factors, but also by different climatological differences. The air pollution priority areas of Mpumalanga, Gauteng and KwaZulu-Natal provinces are home to the majority of coal reserves, mining and steel facilities in South Africa. The combined impact of these anthropogenic sources with other local sources of PM_10_ and different climatic zones is likely to result in spatial variation in PM_10_ concentration levels between the provinces resulting in distinct provincial characteristics of PM_10_, which are not transferable between the provinces. 

Our provincial models were based on few monitoring stations relative to the size of the four provinces. For example Western Cape Province, the largest province among the four provinces (area = 129,462 km^2^), has only 10 operating sites to capture the variability in PM_10_. The lack of sufficient representative monitoring sites to capture intra-province variability in PM_10_ could explain the relative poor performance of the provincial and national models. Previous studies also reported on the limitation of regulatory monitoring networks in capturing small-scale spatial variations of pollutant concentrations due to the sparse distribution of the few monitoring stations [30,31]. 

The site-specific models’ PM_10_ predictions did not differ substantially from the distribution pattern of the observed PM_10_ (Table 3). The site-specific RF models, only using temporal predictor variables, were able to capture the observed temporal variability in PM_10_ better than the national and provincial models. Previous studies in India and Switzerland have explored the association between PM_2.5_ and PM_10_ in co-located sites to impute missing daily PM_2.5_ observations. These studies were able to develop imputation models explaining 89% (Switzerland) and 92% (India) variability in PM_2.5_ [16,17]. These two studies were able to use sufficient PM_10_ and PM_2.5_ measurements at co-located sites to inform their models and then apply these to PM_10_ only sites to impute PM_2.5_. In South Africa, there were insufficient co-located sites to follow this approach. Despite this disadvantage, we were able to explain PM_10_ variance by between 29% and 83% in the site-specific models. 

This finding highlights the paucity of air quality monitoring data in South Africa where only four provinces provided PM_10_ measurements used for this study. Increasing the number of air pollution monitoring sites in South Africa and improving the data capture will provide more power to model more improved and reliable exposure estimates. Nonetheless, the RF variable of importance ranking across the four provinces indicates that chemical transport model estimates of PM_10_ and meteorological variables contributed considerably to explaining ground-level PM_10_ across our study area and study period. 

## 5. Conclusions 

This study compared three models (national, provincial and site-specific) combining spatial, temporal and chemical transport model-based estimates of PM_10_, O_3_ and NO_2_ with observed PM_10_ concentrations to predict missing daily PM_10_ concentrations across 44 monitoring sites in four provinces of South Africa between 2010–2017. Given the extent of air quality monitoring currently conducted in South Africa, the site-specific and provincial models showed a better performance compared to the national models in capturing the variability of ground-level PM_10_. Thus, our study provides evidence that a model constructed with sites from a province is less generalizable to another province. The results of this study, complete time-series of daily PM_10_ concentrations containing a mix between measured and imputed PM_10_ concentrations, will be used in subsequent air pollution exposure studies aimed at informing population health studies in South Africa.

## Figures and Tables

**Table 1:** Spatial and temporal predictors used for random forest models

Variable	Description	Source	Resolution
Population density	Mean population within 1 × 1 km^2^ grid cell	SEDAC	~1 km
Landcover	South Africa National Land Cover 2018 densities (summary of meters within the grid cells by land cover categories of Natural, Built-up, Residential, Agricultural, Industrial)	South Africa Department of Environmental Affairs.	20 m
Light at night	1 × 1 km^2^ Intersected aggregate	VIIRS-DNB	750 m
Impervious Surface	1 × 1 km^2^ Intersected aggregate after removing no data, clouds, shadows data	NOAA	30 m
Elevation	1 × 1 km^2^ intersected aggregate of mean elevation	SRTM Digital Elevation Database	90 m
Roads	Summary of road length distance to nearest road type: major roads and other roads	OpenStreetMap	Lines
Climate zones	Cold interior, Temperate interior, Hot interior, Temperate coastal, Sub-tropical coastal, Arid interior	South Africa Bureau of Standards 2005	6 Zones
Meteorological variables (daily modelled planetary boundary layer height, temperature, precipitation, wind speed, wind direction, relative humidity, vertical velocity	Daily global ECMWF re-analysis estimates	ERA5-reanalysis	10 × 10 km
Modeled Tropospheric estimates of NO_2_, PM_10_, O_3_	Daily Chemical transport model estimate	Chemical transport model Copernicus Atmosphere Monitoring Service (CAMS)	10 × 10 km

Abbreviations: SEDAC (Socioeconomic Data and Applications Center), VIIRS-DNB(Visible Infrared Imaging Radiometer Suite-Day/Night Band), NOAA(National Oceanic and Atmospheric Administration, SRTM (Shuttle Radar Topography Mission), ERA-5 (European Centre for Medium-Range Weather Forecasts Reanalysis 5th Generation).

**Table 2:** Summary of model performance statistics over the period 2010–2017 for the national, provincial and site-specific models showing the range of R^2^, root mean squared error (RMSE) and mean absolute error (MAE) for the years included.

	Model Building			Spatial LOLO CV			Temporal LTO CV			Data Availability	
	R^2^ (Range)	RMSE (Range)	MAE (Range)	R^2^ (Range)	RMSE (Range)	MAE (Range)	R^2^ (Range)	RMSE (Range)	MAE (Range)	No of Unique Sites	Years
**National**	0.77–0.79	12.1–16.76	8.69–11.38	0.11–0.35	17.72–29.47	13.62–23.65	0.77–0.79	12.31–16.43	8.85–11.39	20–44	2010–2017
**Provincial** *											
Mpumalanga	0.73–0.81	14.03–19.35	9.63–12.13	0.39–0.69	22.06–36.21	13.5–29.59	0.73–0.78	13.55–19.21	9.85–12.01	5–17 *	2010–2017
Gauteng	0.49–0.79	10.34–23.36	9.24–16.75	0.26–0.52	19.72–34.25	15.69–29.42	0.52–0.79	15.11–23.43	9.94–16.87	6–18 *	2010–2017
Western Cape	0.29–0.71	6.74–8.73	5.11–6.72	0.35–0.54	7.38–11.22	5.76–8.86	0.44–0.66	6.66–23.29	5.18–17.92	1–11 *	2010–2017
KwaZulu-Natal	0.55–0.79	7.36–9.53	5.29–8.11	0.29–0.57	8.54–19.95	6.95–16.82	0.47–0.78	7.37–10.71	5.46–8	3–6 *	2010–2017
**Site-specific** **											
Beliville	0.42–0.47	5.81–9.16	4.51–7.26	NA	NA	NA	0.45–0.49	5.67–9.02	4.45–7.03	NA	2012, 2013, 2015–2017
Bodibeng	0.54–0.63	16.89–19.42	13.61–15.07	NA	NA	NA	0.57–0.67	16.36–18.91	13.32–14.87	NA	2012–2013
Brackenham	0.41–0.49	8.06–8.95	6.31–7.10	NA	NA	NA	0.46–0.49	7.81–8.95	6.25–7.15	NA	2011, 2015–2017
Booysens	0.45–0.67	22.13–22.82	17.99–20.77	NA	NA	NA	0.5–0.71	22.10–25.74	17.87–20.53	NA	2012,2014
Camden	0.38–0.62	10.64–23.27	8.69–17.85	NA	NA	NA	0.39–0.65	10.29–22.43	9.61–17.15	NA	2013, 2015, 2017
CBD	0.38–0.59	6.35–9.55	4.93–7.45	NA	NA	NA	0.41–0.64	6.28–9.23	4.98–7.21	NA	2011–2013, 2015–2017
City Hall	0.45	10.29	7.69	NA	NA	NA	0.48	9.78	7.43	NA	2010
Elandsfontein	0.39–0.52	11.72–12.49	9.38–9.68	NA	NA	NA	0.45–0.57	11.17–11.79	8.99–9.38	NA	2016–2017
Ermelo	0.48–0.76	9.20–18.96	7.69–15.31	NA	NA	NA	0.51–0.77	9.12–19.98	7.54–13.89	NA	2010–2016
Etwatwa	0.63	24.03	18.74	NA	NA	NA	0.69	23.78	18.56	NA	2012
Ferndale	0.68–0.74	3.63–5.42	2.84–3.92	NA	NA	NA	0.65–0.77	3.49–5.38	2.76–3.88	NA	2010–2012
Foreshore	0.32–0.49	5.29–9.76	4.1–7.22	NA	NA	NA	0.33–0.49	5.27–9.58	4.13–7.08	NA	2011–2013,2015–2017
Gangles	0.48–0.74	11.86–13.4	9.22–10.11	NA	NA	NA	0.51–0.75	11.23–11.88	8.96–9.71	NA	2010, 2011, 2013,2014
Germiston	0.42	19.65	14.96	NA	NA	NA	0.44	19.07	14.79	NA	2011
George	0.55–0.56	7.09–8.41	5.49–6.56	NA	NA	NA	0.58	6.95–8.12	5.39–6.34	NA	2010, 2013
Goodwood	0.46–0.57	6.77–8.78	5.26–8.24	NA	NA	NA	0.49–0.59	6.60–8.49	5.29–7.80	NA	2011–2012, 2014–2016
Grootvlei	0.41–0.44	10.76–11.32	8.70–8.87	NA	NA	NA	0.42–0.49	10.65–11.12	8.63–8.82	NA	2011, 2013
Hendrina	0.39–0.71	11.12–17.02	8.32–13.62	NA	NA	NA	0.43–0.74	11.18–16.56	8.36–12.96	NA	2010–2012,2015–2016
Middleburg	0.67–0.81	7.81–19.25	6.08–14.73	NA	NA	NA	0.70–0.82	7.49–18.63	5.92–14.25	NA	2010–2016
Olievenhoutbosch	0.57	34.23	27.01	NA	NA	NA	0.59	34.16	26.98	NA	2012
Orange Farm	0.45–0.69	10.78–19.81	8.57–15.56	NA	NA	NA	0.49–0.71	10.23–19.49	8.28–15.62	NA	2010,2017
Rosslyn	0.55–0.61	5.91–11.49	4.77–9.30	NA	NA	NA	0.52–0.67	5.86–11.05	4.47.8.93	NA	2012–2014
Secunda	0.63–0.77	7.73–25.21	5.86–19.96	NA	NA	NA	0.67–0.77	7.47–24.64	5.75–19.7	NA	2010–2013
Witbank	0.72–0.83	9.21–22.33	7.63–17.27	NA	NA	NA	0.73–0.83	8.79–21.87	7.34–16.75	NA	2010,2013–2016
Komati	0.45–0.83	8.52–28.02	6.61–21.51	NA	NA	NA	0.46–0.84	8.29–27.11	6.5–20.91	NA	2011–2012,2014–2017
Leandra	0.29–0.36	6.63–14	4.86–10.38	NA	NA	NA	0.35–0.4	6.35–13.64	4.81–10.31	NA	2011–2012
Newtown	0.43	22.07	17.52	NA	NA	NA	0.47	21.68	17.27	NA	2012
Phola	0.54–0.65	22.44–28.89	17.83–22.55	NA	NA	NA	0.57–0.65	22.02–28.88	17.48–22.72	NA	2013–2014,2016–2017
Stellenbosch	0.35–0.56	6.34–7.31	4.85–5.67	NA	NA	NA	0.37–0.61	6.26–7.14	4.83–5.62	NA	2012–2013
Tableview	0.36–0.4	5.63–7.04	4.43–5.81	NA	NA	NA	0.38–0.43	5.54–7	4.31–5.6	NA	2011–2013
Tembisa	0.71	17.78	14.09	NA	NA	NA	0.73	17.35	13.89	NA	2011
Thokoza	0.56	41.30	29.22	NA	NA	NA	0.57	40.25	28.76	NA	2011
Wallacedene	0.47–0.51	5.53–11.26	4.28–8.9	NA	NA	NA	0.47–0.54	5.52–10.82	4.29–8.69	NA	2012, 2015–2017
Wattville	0.52	39.10	29.09	NA	NA	NA	0.57	37.16	28.57	NA	2012
Club	0.59–0.67	11.01–14.87	8.76–11.86	NA	NA	NA	0.62–0.69	10.7–14.88	8.55–11.99	NA	2012–2014, 2016–2017
Ekandustria	0.46–0.59	11.14–16.83	8.88–13.09	NA	NA	NA	0.50–0.64	10.58–16.43	8.5–12.83	NA	2013–2014
Embalenhle	0.56–0.73	16.48–22.18	11.34–14.69	NA	NA	NA	0.59–0.73	13.31–22.18	11.03–17.86	NA	2012,2014,2016–2017
Verkykkop	0.44–0.49	6.63–9.71	5.53–7.88	NA	NA	NA	0.47–0.48	6.56–9.49	5.33–7.72	NA	2013,2016–2017
Randwater	0.32–0.73	12.99–15.99	9.82–15.83	NA	NA	NA	0.36–0.75	12.08–15.63	9.57–12.19	NA	2013–2017
Esikhaweni	0.43–0.58	9.07.9.45	7.36–7.4	NA	NA	NA	0.44–0.60	8.95–9.35	7.17	NA	2016–2017
Chicken Farm	0.44	13.14	10.44	NA	NA	NA	0.48	12.71	10.21	NA	2017
Kwazamokuhle	0.65	18.10	14.44	NA	NA	NA	0.67	17.10	13.84	NA	2017
Kriel Village	0.62	17.27	13.55	NA	NA	NA	0.66	16.89	13.41	NA	2017
Bosjesspruit	0.51	13.05	10.44	NA	NA	NA	0.55	12.58	10.27	NA	2017

* The provincial models included all possible sites with PM_10_ observation; ** The sites models included the monitoring stations with at least 70% annual PM_10_ observation. NA: Not applicable. These are individual site models—Spatial cross-validation (CV) cannot be perform for models with less than two sites. LOLO: Leave one location out spatial cross-validation; LTO: Leave time out temporal cross-validation. Range: The minimum and maximum values of the statistics metrics from the models across 2010.

**Table 3:** Range of the observed versus predicted PM_10_ concentrations (in µg/m^3^) for the 3 different models (National, Provincial and Site-specific) averaged over all sites and years (2010–2017) by province for the mean, standard deviation (SD) and 5th, 25th, 50th, 75th and 95th percentiles).

Province		Mean	SD			Percentiles		
		µg/m^3^	µg/m^3^	5	25	50	75	95
Mpumalanga	Observed	35.70–50.90	17.70–29.10	9.30–15.30	21.40–30.30	32.90–46.20	47.70–71.20	68.20–102.80
	National	34.60–48.60	6.30–11.10	23.70–34.20	29.20–41.10	34.30–47.80	39.50–56.80	45.70–66.50
	Provincial	34.20–46.30	10.40–17.40	17.10–24.70	24.90–33.60	32.20–44.30	42.30–60.40	53.00–75.80
	Site-specific	35.70–52.00	11.40–19.50	18.60–26.10	26.80–37.10	34.30–49.80	43.30–66.90	55.50–85.40
Gauteng	Observed	53.40–58.30	28.40–31.30	16.20–20.30	31.10–35.20	47.50–52.10	71.10–77.10	107.60–115.00
	National	36.30–41.60	10.20–12.90	21.30–24.40	27.00–31.00	34.80–40.70	44.60–52.00	54.00–62.40
	Provincial	52.90–59.40	16.90–17.90	30.80–35.50	40.30–45.40	50.20–56.50	66.10–73.30	81.20–90.00
	Site-specific	53.00–58.40	17.40–19.70	29.30–33.50	37.90–43.10	49.70–54.80	65.60–72.30	84.70–93.20
Western Cape	Observed	19.50–26.70	8.10–11.60	8.50–12.70	13.40–18.70	18.50–25.20	24.30–33.30	35.00–48.10
	National	31.90–49.10	7.10–11.20	22.00–35.90	26.00–41.00	29.90–46.80	36.60–55.40	45.20–71.60
	Provincial	20.00–28.00	39.00–5.50	13.50–20.40	16.70–24.10	20.00–28.00	22.70–31.80	26.90–37.10
	Site-specific	19.50–26.70	4.80–6.60	11.80–17.90	15.90–21.80	18.80–26.20	22.40–30.70	28.00–38.40
KwaZulu-Natal	Observed	24.20–29.80	11.01–14.01	9.50–13.50	15.90–20.01	22.10–26.60	30.70–37.10	45.70–56.60
	National	31.60–43.80	8.20–12.90	21.10–28.40	24.50–33.40	29.00–40.40	37.60–53.00	47.60–66.00
	Provincial	23.90–32.90	5.20–9.50	15.60–21.60	19.20–25.90	22.50–31.60	27.10–39.40	35.40–49.50
	Site-specific	24.20–30.50	6.01–10.02	15.30–19.70	19.10–23.30	23.00–28.30	28.00–36.00	36.00–50.80

## Data Availability

Data sharing not applicable.

## Supplementary Materials

The following are available online at https://www.mdpi.com/1660-4601/18/7/3374/s1, Figure S1: Mpumalanga province random forest variable of importance, Figure S2: Gauteng province random forest variable of importance, Figure S3: Western Cape province random forest variable of importance, Figure S4: KwaZulu-Natal province random forest variable of importance, Figure S5: Site-specific model’s random forest variable of importance.
