# Supplementary material for: Comparing Methods to Impute Missing Daily Ground-Level PM10 Concentrations between 2010–2017 in South Africa
Source: Int J Environ Res Public Health. 2021 Mar 24;18(7):3374. doi: 10.3390/ijerph18073374 (PMC8037804; doi:10.3390/ijerph18073374)
Supplement: Supplementary file 1 [file ijerph-18-03374-s001.pdf]

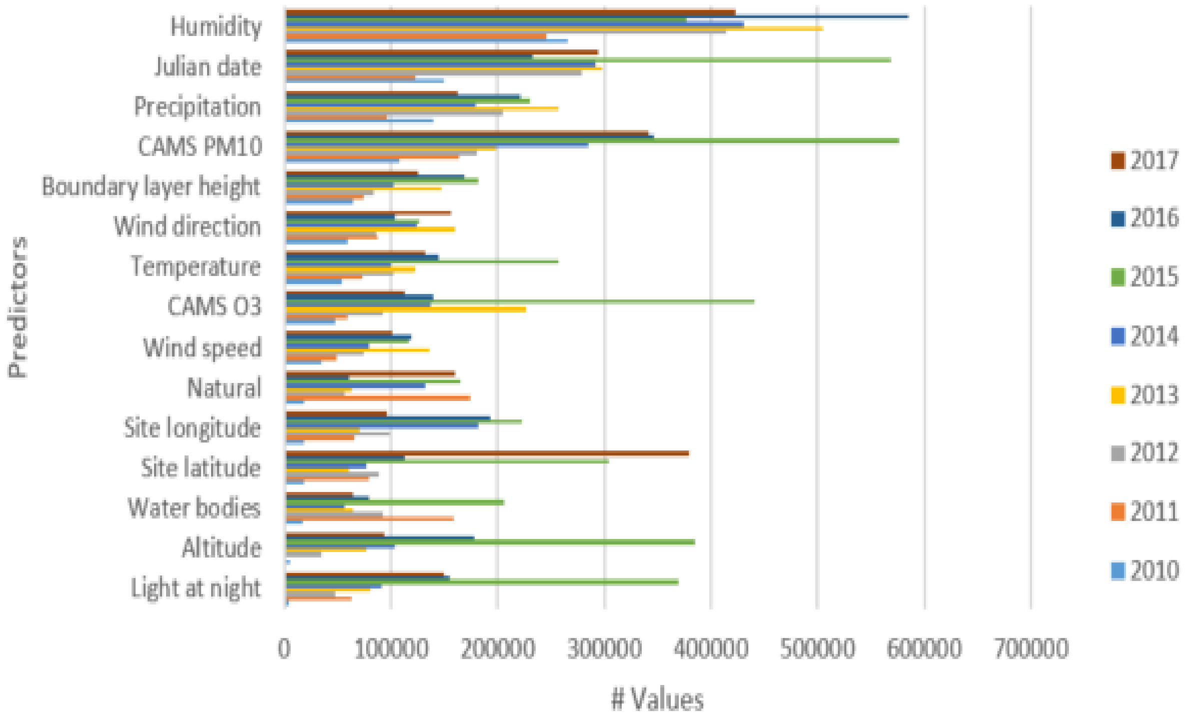

Figure S1: Mpumalanga province Random Forest Variable of Importance

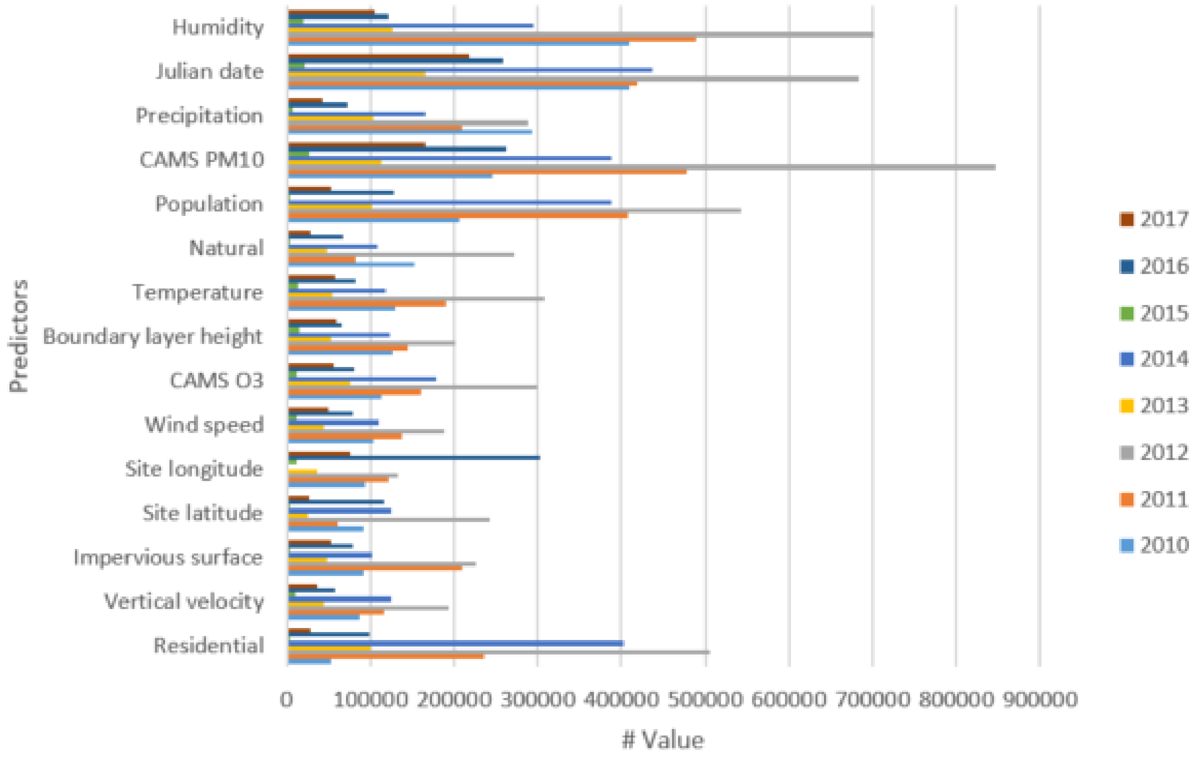

Figure S2: Gauteng province Random Forest Variable of Importance

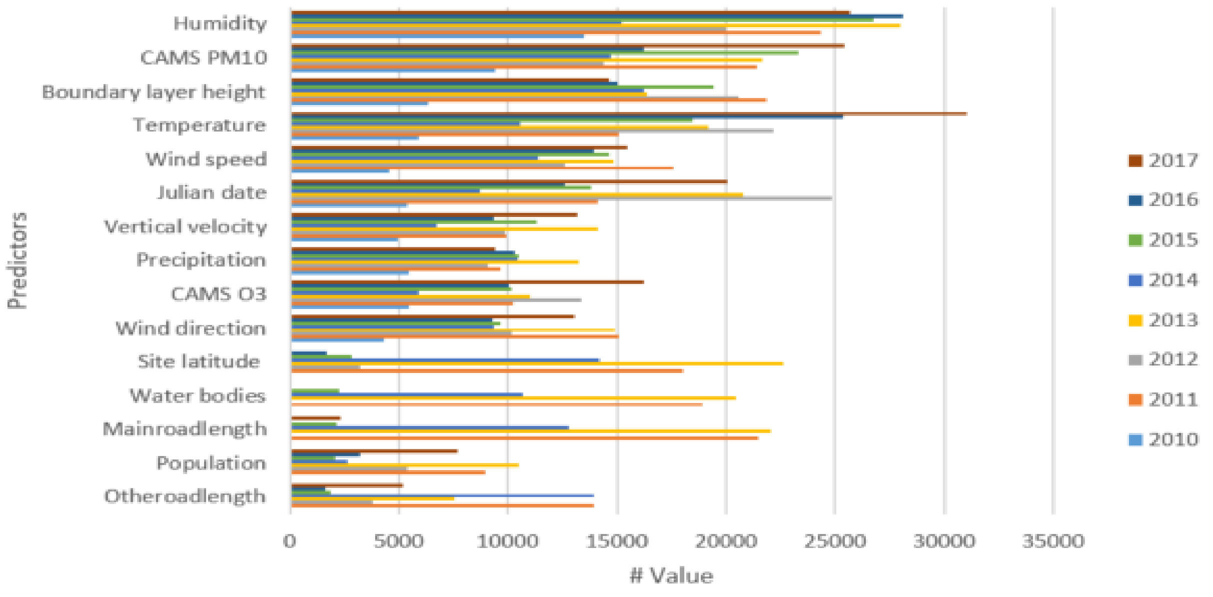

Figure S3: Western Cape province Random Forest Variable of Importance

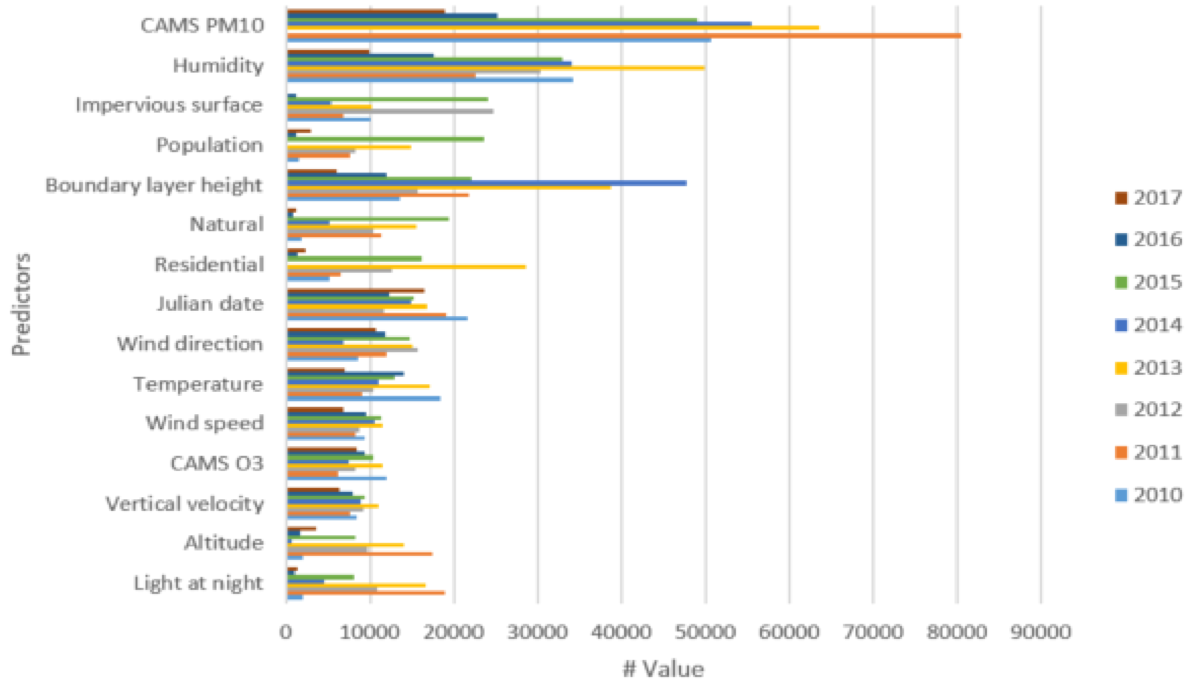

Figure S4: KwaZulu-Natal province Random Forest Variable of Importance

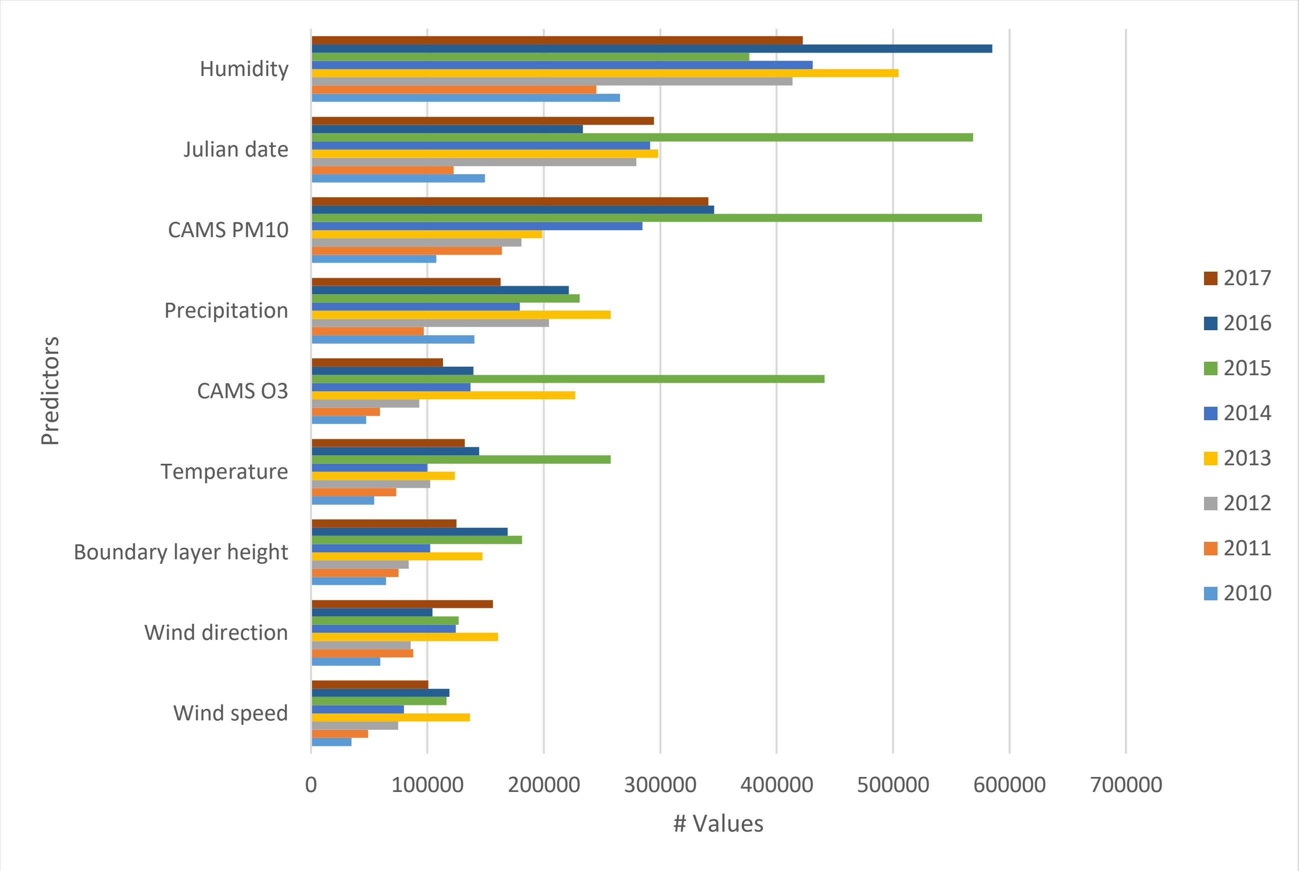

Figure S5: Site-Specific Models Random Forest Variable of Importance
